# Adjacent Segment Infection after Lumbar Fusion: A Case Report and the Literature Review

**DOI:** 10.1155/2020/2163909

**Published:** 2020-01-20

**Authors:** Wenlong Wang, Zheng Liu, Sijun Wu

**Affiliations:** Department of Orthopedics, Peking University Shougang Hospital, Shijingshang District, Beijing 100144, China

## Abstract

**Introduction:**

Adjacent segment infective spondylodiscitis is a rare type of surgical spinal infection after lumbar fusion with few reports. We report a further case of adjacent segment infection after three-level lumbar fusion managed successfully with anti-infective therapy and revision surgery. *Case Description*. A clinical case of a 69-year-old female with multilevel lumbar degenerative disease received three-level fusion. The leading preoperative symptoms were relieved after decompression surgery. However, severe back pain recurred and prompted her to be rehospitalized 2 months later. The signal of spondylitis and discitis at the adjacent segment was detected by magnetic resonance imaging (MRI). No bacteria were identified despite blood cultures being taken before antibiotic treatment. After a long-term anti-infective therapy with vancomycin, the patient gained symptom relief and was discharged home. However, the patient complained of severe back pain again after long-term oral antibiotic treatment and was rehospitalized 6 months after surgery. The computed tomography (CT) scan showed obvious bony endplate destruction at the adjacent segment space. The patient received a debridement operation and autologous iliac bone graft. The infective inflammatory markers were controlled, and the infective space achieved fusion finally.

**Conclusion:**

Adjacent segment space infection is a rare reported complication that occurs after spinal fusion surgery. Conservative antibiotic therapy may not control the infection completely, and disc space debridement and autologous iliac bone graft can achieve ultimate fusion and a satisfactory outcome.

## 1. Background

Postoperative surgical site infection (SSI) is a common and risky complication of spinal surgery. Incidence of SSI in instrumented spinal surgery has been reported to range from 2.2 to 20% [1]. However, the definite incidence of adjacent segment infection (ASI) after instrumented spinal surgery has not been reported. ASI after surgery of spondylodiscitis is considered as a rare complication (1.94%) via a large cohort study [2]. ASI in uninfected spinal surgery should be rarer, and in fact, only four case reports introduced it in conventional spinal fusion surgery [3–6]. In this present case, the patient developed ASI two months after the initial instrumented lumbar fusion surgery and achieved satisfactory outcome via debridement operation and autograft bone implantation fusion after failed conservative treatment.

## 2. Case Presentation

**Figure 1:**
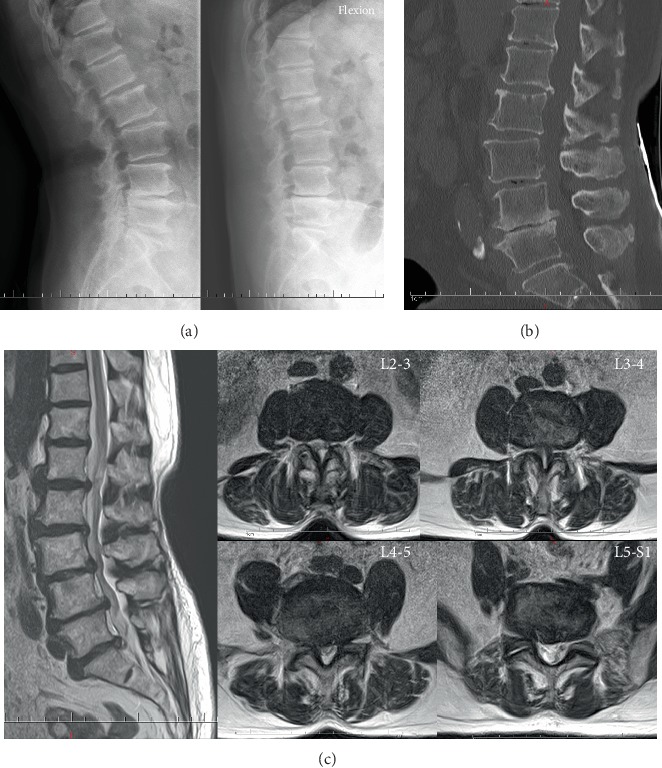
Flexion and extension lateral lumbar X-ray radiograph demonstrating segmental dynamic instability at L2-3 and L3-4 (a). Sagittal lumbar CT showing intervertebral space collapse and osteophyte at L4-5 and “gas sign” at L3-4 and L4-5 discs (b). Sagittal and axial T2-weighted lumbar MRI revealing serious central canal stenosis at L3-4, lumbar disc herniation and right lateral recess stenosis at L4-5, and moderate canal stenosis in L2-3 (c).

A 69-year-old female complained of intermittent right leg radicular pain in the past two years and which became worse with numbness in the recent four days. Moreover, the patient also had severe intermittent claudication, and the claudication distance was only about 20 meters. The decreased muscle force of the right quadriceps femoris and extensor hallucis longus and hypesthesia in the bilateral dorsal foot skin were found through physical examination. The Lasegue sign of the right leg was positive, and the bilateral Babinski signs were negative. The flexion and extension lateral lumbar radiographs showed segmental dynamic instability at L2-3 and L3-4 (Figure 1(a)). Sagittal computed tomography (CT) showed multilevel disc degeneration, severe osteophyte at L4-5, and L3 spondylolisthesis relative to upper and lower vertebra (Figure 1(b)). Magnetic resonance imaging (MRI) revealed serious central stenosis at L3-4 and lumbar disc herniation and right lateral recess stenosis at L4-5 (Figure 1(c)). White blood cell (WBC) count and blood chemistry, C-reactive protein (CRP), and erythrocyte sedimentation rate (ESR) were all unremarkable during hospitalization.

**Figure 2:**
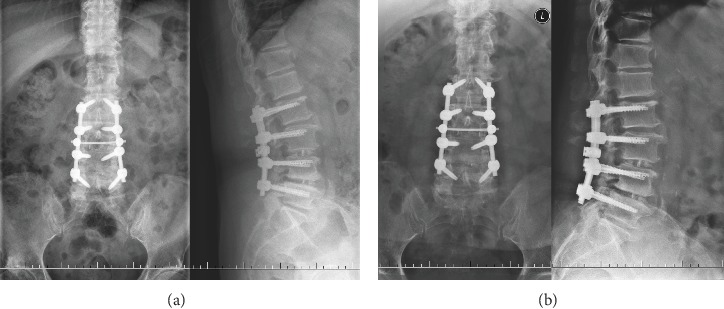
Postoperative anterior-posterior and lateral lumbar radiographs (a). Anterior-posterior and lateral lumbar radiographs of one-month follow-up (b).

Laminectomy of L4 and right-sided hemilaminectomy of L5 were performed to decompress the right L4, L5 nerve root, and dural sac. Discectomy and intervertebral fusion were done in L3-4 and L4-5 levels, and bilateral facet joint fusion of L2-3 was performed due to segmental dynamic instability of L2-3. L2 to L5 were fixed by rigid instruments (DePuy Synthes, West Chester, PA, USA). The right leg pain visual analogue scale (VAS) had decreased significantly the first day after surgery. Celecoxib was given to relieve low back pain. The patient was discharged home after she was able to walk independently without difficulties with a Boston brace (ten days after the surgery). The postoperative and one-month follow-up's lumbar radiographs showed no abnormal findings (Figure 2).

**Figure 3:**
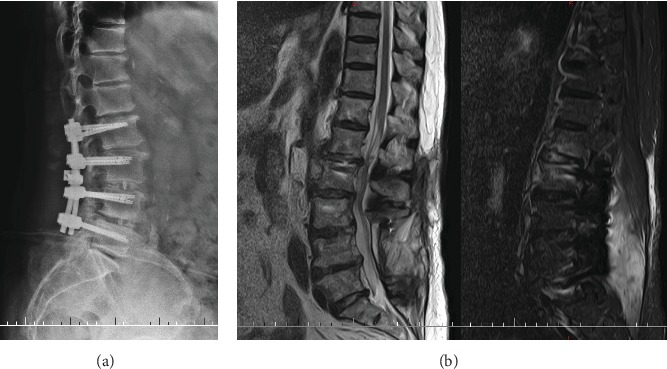
Lateral lumbar radiograph of second hospitalization showing no significant changes at the adjacent L1-2 segment comparing with one-month follow-up (a). Sagittal MRI T2-weighted and short-tau inversion recovery (STIR) images of second hospitalization showing inflammatory edema signal in intervertebral disc and bone marrow below the endplates of L1-2 (b).

Two months after the surgery, however, the patient returned to our department with serious throbbing low back pain. The physical examination did not reveal any dysfunction of lumbar and sacral nerves. The surgical wound had healed completely. And there was a little percussion pain over the spinous process of upper instrumented vertebrae. The blood laboratory test was indicative of bacterial infection, WBC count 7.5∗10^9^/L, neutrophile granulocyte percentage (NEU) 87.9%, ESR 16 mm/h, CRP 87.25 mg/L, and procalcitonin (PCT) 0.049 *μ*g/L. Although the location of the infection and pathogenic bacteria were unclear, the empirical antibiotic vancomycin was prescribed after vein blood culture. However, the blood culture did not show any bacteria growth a few days later. The spondylodiscitis signal of adjacent segment in lumbar MRI further confirmed our hypothesis of infection of the lumbar spine (Figure 3(b)).

**Figure 4:**
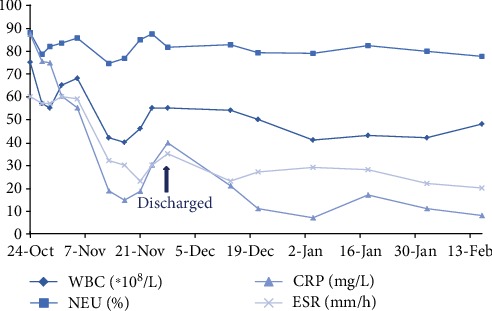
Main blood inflammatory indexes ranged from second admission to last out-patient review. WBC: white blood cell count; NEU: neutrophile granulocyte percentage; CRP: C-reactive protein; ESR erythrocyte sedimentation rate.

The low back pain was relieved by vancomycin anti-infective therapy and celecoxib. After 5 weeks intravenous vancomycin anti-infective treatment, the patient was discharged home and we advised our patient to proceed with orally administered cefdinir. The blood laboratory tests were done periodically, and the main inflammatory index changing curves are shown in Figure 4.

**Figure 5:**
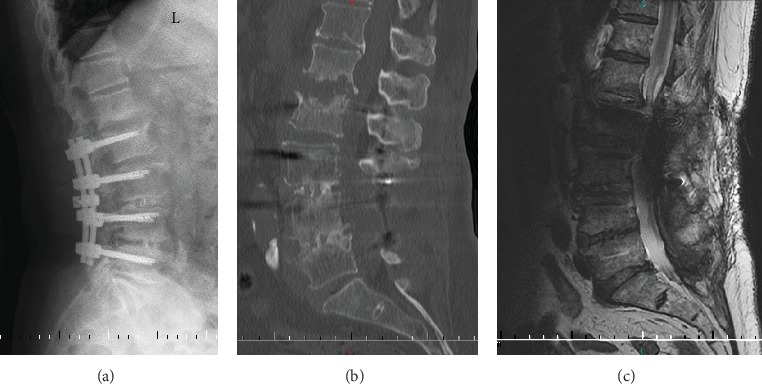
Lateral lumbar radiograph of third hospitalization showing the destructive endplates at the L1-2 space (a). Sagittal CT of third hospitalization showing spondylodiscitis and destruction of bony endplates at the L1-2 segment (b). Sagittal MR T2-weighted imaging of third hospitalization revealing spondylodiscitis at the L1-2 segment (c).

Within 20 days after the last review, the patient presented again with exacerbation of throbbing low back pain. The blood laboratory test suggested that the spondylodiscitis in L1-2 was uncontrolled, WBC count 10.1∗10^9^/L, NEU percentage 89.9%, ESR 30 mm/h, CRP 38.40 mg/L, and PCT 0.047 *μ*g/L. Vancomycin and cefoperazone were combined to cover both Gram-positive cocci and Gram-negative bacilli, and oral metronidazole was used to cover the anaerobe as well. Moreover, the radiographic images revealed serious spondylodiscitis and bony endplate destruction at the adjacent L1-2 segment (Figure 5).

**Figure 6:**
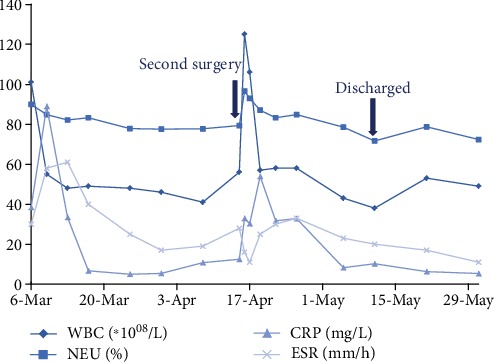
Main blood inflammatory indexes ranging from third hospitalization to recent out-patient review. WBC: white blood cell count; NEU: neutrophile granulocyte percentage; CRP: C-reactive protein; ESR: erythrocyte sedimentation rate.

**Figure 7:**
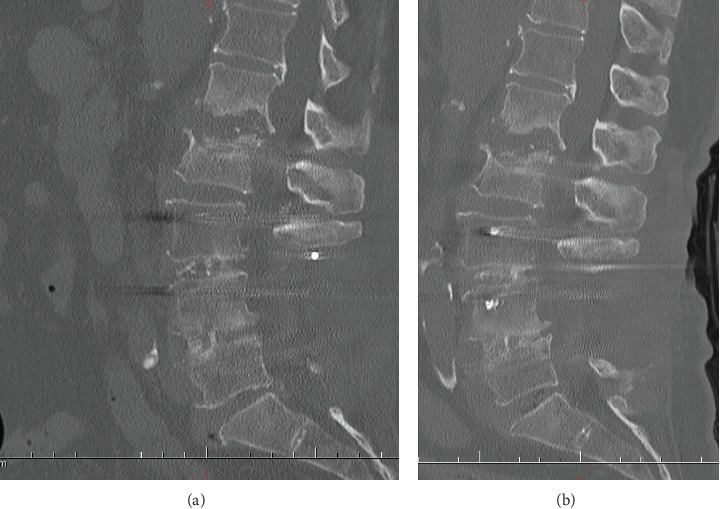
Sagittal lumbar CT after two-week anti-infective therapy (a). Sagittal lumbar CT after one-month anti-infective therapy showing little changes at the L1-2 space comparing with previous lumbar CTs during third hospitalization (b).

The combined anti-infective therapy continued for a week, and the serious low back pain relieved quickly with blood inflammatory indexes dropping significantly. Single intravenous cefoperazone was used sustainably, and radiographic examinations were taken to observe the destructive extent of L1-2 segmental spondylodiscitis. The patient needed to keep strictly on her bed to avoid infective space collapse. After five-week continuous anti-infective therapy, the main blood inflammatory indexes dropped to the normal level (Figure 6), with slight residual symptom. However, the spondylodiscitis and bony endplate destruction at the L1-2 space had little changes (Figure 7).

**Figure 8:**
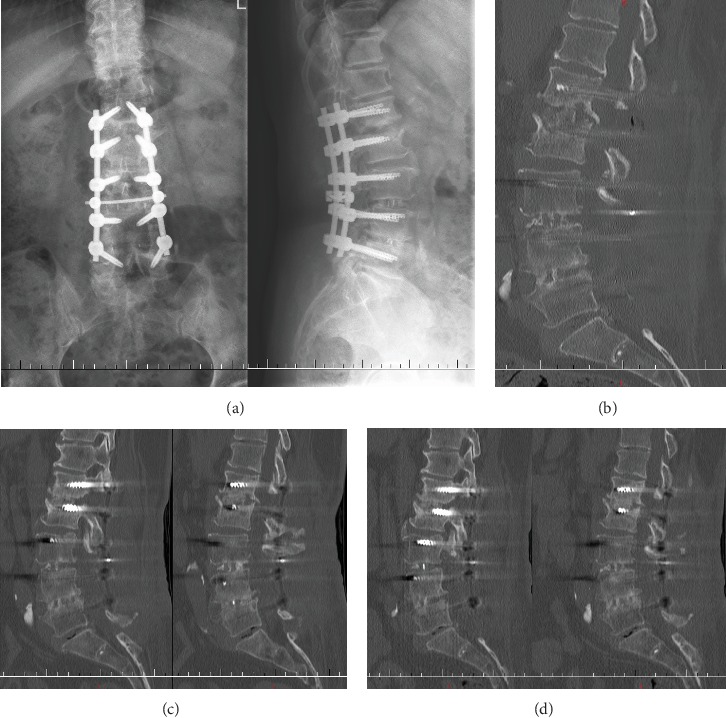
Anterior-posterior and lateral lumbar radiographs 1 week after revision. The upper instrumented level was extended to L1 (a). Sagittal lumbar CT 1 week after revision. The infective tissues at the L1-2 space were debrided and then an iliac crest autograft was implanted (b). Sagittal lumbar CT 6 weeks after revision. The surgical space tended to fuse with well-placed instruments (c). Sagittal lumbar CT 6 months after revision showed satisfactory fusion sign in the infective space (d).

The patient then underwent posterior intervertebral debridement and posterior superior iliac crest autograft implant at the L1-2 space; the initial pedicle screws in L2 were taken out for high temperature sterilization and reimplanted. And the upper instrumented level was extended to L1 (Figures 8(a) and 8(b)). The wound drainage was pulled out after continuous negative bacterial culture of drainage liquid. The patient had satisfactory symptom relief and significant inflammatory indexes decreased (Figure 6). The debrided tissues from L1-2 did not show tumor, tuberculosis, and fungal infection through pathologic examination, and the bacterial culture did not show any bacteria growth. The patient was able to walk independently with the Boston brace (one week after revision surgery). The intravenous cefoperazone was used sustainably for four weeks after the second surgery, and then the patient was discharged.

The patient came back to the out-patient department for review every two weeks. The inflammatory indexes almost dropped to normal (Figure 6). The six-week follow-up's CT scan showed that the destructive space was closed to fusion (Figure 8(c)). The patient did not complain of obvious symptoms any more.

## 3. Discussion

The first ASI case reported by Kulkarni and Hee [3] introduced a mild paresthesia patient caused by a C4-5 level epidural abscess after C5-C7 fusion. This was also the unique ASI case reported in the cervical spine. Siam et al. [2] reported the currently largest ASI series, and postoperative ASI was considered as a rare complication (23/1187). However, all these ASI cases occurred after surgical treatment of spondylodiscitis. For common uninfective spinal surgeries, this complication incidence could be rarer. According to Siam et al.'s study, the most commonly involved level was L3-4, the next were T12-L1 and L2-3. Nagoshi et al. [6] reported three ASI cases which involved T11-12, L1-2, and L2-3. A case reported by Xin and He [5] occurred at level L3-4. Formica et al. [7] and Lange et al. [4] reported two ASI cases which occurred in the thoracic spine. The ASI could occur at both long segment fusion and short segment fusion, and all infective segments in these reported cases were proximally adjacent to fusion levels.

The potential causes of ASI are still unknown; Kulkarni and Hee [3] hypothesized an accidental inoculation of bacteria into the disc space intraoperatively by a contaminated radiological marker, and hematogenous spread may be the second possibility. The adjacent segment spondylodiscitis case reported by Formica et al. [7] was considered to be caused by hematogenous infection. The pedicle screw instrument which allowed the pathogen to get into the vertebral body or directly penetrated the endplate was considered as the possible reason [4, 5]. The pedicle screw close to the endplate could disrupt the arterial network of the endplate, leading to the formation of septic embolus and spondylodiscitis [6]. Nagoshi et al. [6] also thought the pathogenesis of ASI was just like the adjacent segment degeneration (ASD), a result of the concentrated stress at the adjacent level of spinal fusion. The disruption of the annulus fibrosus in an adjacent segment was regarded as a kind of infective route by some authors [5]. Bacteria could tend to remain at the endplate due to a disturbance in blood flow. In our patient, the upper pedicles were located closely to the endplate of L2, and this could be the possible reason. The cannulated screws we used may create an “immune escape” space inside the screws according to Lange et al.'s theory [4]. Moreover, the hematogenous septic embolus was considered as the infective approach, because incomplete intestinal obstruction had been diagnosed in this patient before the second admission.

The treatment strategies of ASI were similar to SSI. In patients whose infection had been diagnosed early, they can be cured through conservative antibiotic treatment [5, 6]. In these publications, the most common pathogenic bacteria were *Staphylococci* [4, 6, 7]. *Serratia* was only reported by Kulkarni and Hee [3]. However, Xin and He [5] did not use intervertebral disc puncture for microbiological examination to avoid disc environment destruction and spreading of the infection. For the same reason, we did not puncture the disc in the early period of infection. Even in the disc destruction period, we were not able to culture as a result of the effectiveness of anti-infective therapy. The long-term and high-dose antibiotics made all blood or disc tissue cultures negative, and in the last, we did not confirm the pathogenic bacteria in this case.

The type and duration of antibiotic therapy varied in the literatures. However, antibiotic administration over 6 weeks intravenously and 3 months orally was recommended in most reports [8, 9]. Even after surgical debridement and instrumentation removal, 4-6 weeks of intravenous antibiotic therapy and several weeks of oral therapy were necessary and reported to result in lower recurrence rate [10, 11]. *Staphylococcus* was reported as the most common pathogen in deep or superficial SSI [9, 12]. Therefore, we administered vancomycin intravenously as a first line treatment and later on switched to oral antibiotic treatment. The intravenous antibiotic therapy was stopped after symptom relief and significant improvement of markers of inflammation. However, we noted insufficient improvement of clinical symptoms after empirically applied antibiotic treatment and conservative management. The destroyed segment had resulted in instability and severe symptoms. The indication for surgical revision is based on the failure of conservative treatment and occurrence of neurological symptoms caused by instability [9].

In most reported ASI cases, surgical debridement and instrumented fusion was considered a rewarding last option [2, 3, 6, 7]. We concluded the surgical indications were epidural abscess compression, segmental instability due to serious bony destruction, severe kyphosis due to intervertebral space collapse, medically intractable pain, and failed anti-infective treatment or antibiotic resistance. In this patient, debridement and instrumented fusion of L1-2 was performed to clear infective tissues and reconstruct segmental stability. Due to the fact that the infective region was limited to the upper adjacent segment, the surgical equipment involved in the infective or noninfective region was used separately. The instrument which had been exposed to the infective tissue had to be removed and replaced by a new one. High temperature sterilization of surgical instruments and implants applied intraoperatively would have been another cost saving option. The surgical flushing solution of the infective tissue was strictly prohibited to contact with the noninfective tissue. Moreover, sustained anti-infective therapy may result in antimicrobial resistance and recurrence of infection. Various spinal fusion techniques have been reported, and both anterior and posterior approaches were able to achieve excellent long-term outcomes [6, 7]. In the future, we may choose the oblique lumbar intervertebral fusion (OLIF) for debridement and intervertebral fusion just like its use in the revision surgery of ASD [13]. The initial instrument was not recommended to be removed unless the screws were loose or connected to the infective space [14]. Removal of implants may result in loss of correction, spinal instability, and clinical symptoms, such as back pain, radicular pain, or neurologic deficits [12]. Although all foreign materials including devascularized autograft may decrease antibiotic efficacy [15], the tricortical autograft which could provide osteogenic, osteoinduction, and osteoconduction properties was considered as the gold standard for fusion [16]. Autologous iliac crest bone graft was the most common graft material for infective bone defects and intervertebral fusion [2–4, 6, 17]. We chose the autograft from the posterior superior iliac crest because of the identical operative position during revision surgery.

Further prospective multicenter investigations are required to confirm our findings. Our findings reported herein should be interpreted cautiously.

## 4. Conclusion

We report a rare site infection after lumbar fusion surgery. The potential causes of ASI are still unclear, and postoperative back pain caused by ASI is easy to be ignored. The definitive bacterial evidence should be obtained as soon as possible for sensitive antibiotic treatment before empirical anti-infective therapy. The laboratory infective index and lumbar radiography should be reviewed regularly. A one-stage infective space debridement and autologous bone graft is recommended for fusion and segmental stability. The internal instruments should be retained to achieve early stability and ultimate fusion.
